# Spatial patterns of *Fasciola hepatica* and *Calicophoron daubneyi* infections in ruminants in Ireland and modelling of *C. daubneyi* infection

**DOI:** 10.1186/s13071-018-3114-z

**Published:** 2018-09-29

**Authors:** Amalia Naranjo-Lucena, María Pía Munita Corbalán, Ana María Martínez-Ibeas, Guy McGrath, Gerard Murray, Mícheál Casey, Barbara Good, Riona Sayers, Grace Mulcahy, Annetta Zintl

**Affiliations:** 1 School of Veterinary Medicine, University College Dublin, Belfield, Dublin 4, Ireland; 2 Teagasc AGRIC, Moorepark, Fermoy, Co. Cork, Ireland; 3 UCD Centre for Veterinary Epidemiology and Risk Analysis, Belfield, Dublin 4, Ireland; 4 RVL Division, Department of Agriculture, Food and Marine, Backweston, Celbridge, Co. Kildare Ireland; 5 Teagasc AGRIC, Athenry, Co Galway Ireland

**Keywords:** *Calicophoron daubneyi*, *Fasciola hepatica*, co-infection, Kernel density, Machine Learning, Risk factors, Risk mapping, Prediction

## Abstract

**Background:**

*Fasciola hepatica* has always represented a threat to Irish livestock because the Irish climate is highly suitable for the main local intermediate host of the parasite, the snail *Galba truncatula*. The recent clinical emergence of infections due to *Calicophoron daubneyi* has raised the question of whether the two parasites, which share a niche during part of their life-cycles, interact in some way. Here, we used geographical information systems (GIS) to analyse the distribution of both parasites in cattle and sheep. We also developed the first predictive model of paramphistomosis in Ireland.

**Results:**

Our results indicated that, in cattle, liver fluke infection is less common than rumen fluke infection and does not exhibit the same seasonal fluctuations. Overall, we found that cattle had a higher likelihood of being infected with rumen fluke than sheep (OR = 3.134, *P* < 0.01). In addition, infection with one parasite increased the odds of infection with the other in both host species. Rumen fluke in cattle showed the highest spatial density of infection. Environmental variables such as soil drainage, land cover and habitat appeared to be the most important risk factors for *C. daubneyi* infection, followed by rainfall and vegetation. Overall the risk of infection with this parasite was predicted to be higher in the west of the country.

**Conclusions:**

This study shows differences between the infection rates and spatial patterns of bovine and ovine infections with *F. hepatica* and *C. daubneyi* in Ireland. Whether the reasons for this are due to susceptibility, exposure and/or management factors is yet to be determined. Furthermore, the rumen fluke model indicates distinct risk factors and predicted distribution to those of *F. hepatica*, suggesting potential biological differences between both parasite species.

## Background

The helminth parasite *Fasciola hepatica* (the liver fluke) is the causative agent of fasciolosis, which is of high economic importance in ruminants. The parasite can also infect a wide range of other mammals. Fasciolosis causes annual losses estimated at around €2.5 billion to livestock and food industries worldwide and losses of about €90 million every year in Ireland [1]. Economic losses are mainly due to decreased meat and milk production, reduced fertility and increased rates of liver condemnation [2–5]. Moreover, severe acute infections may cause death as a result of haemorrhage and liver damage, particularly in lambs.

Increasing prevalence of the rumen fluke, *Calicophoron daubneyi*, and sporadic clinical cases of paramphistomosis have been reported since the late 2000s in Ireland and the UK [6–11], leading to the suggestion that *C. daubneyi* is now the dominant rumen fluke species in Europe [12–16] with significant clinical importance in ruminants in Europe [17]. However, clinical paramphistomosis is still relatively rare in Ireland, and chiefly associated with the feeding activity of excessive numbers of immature stages attached to the mucosal wall of the small intestine [18]. In contrast, even heavy infections with adult rumen flukes are generally benign.

The life-cycle of *C. daubneyi* shares features with that of *F. hepatica,* involving the same intermediate and definitive hosts, although there are some important differences. Ruminants become infected by the ingestion of metacercariae (cysts) on pasture. Larvae excyst in the abomasum as the cyst wall is digested, and travel to the duodenum and jejunum. Newly excysted juveniles (NEJs) of *C. daubneyi* attach to the intestinal mucosa and feed on blood for about three to six weeks. Afterwards, they leave the small intestine to migrate to the rumen, where they attach to the wall by their oral sucker and feed on ingesta [19, 20]. NEJs of *F. hepatica*, on the other hand, do not remain in the intestine, but migrate directly through the intestinal wall and peritoneum to the liver. Here they burrow through the parenchyma for a number of weeks before settling in the bile ducts [21]. Both parasites mature approximately three months after being ingested, and lay eggs that are passed with the host’s faeces [22]. Fluke eggs on pasture hatch into miracidia (larvae) that develop from sporocysts to rediae in the snail intermediate hosts (in Ireland chiefly represented by *Galba truncatula*). Eventually cercariae are released and encyst as metacercariae on vegetation.

As both trematodes significantly overlap in their host range and geographical distribution, we sought to analyse possible interactions and/or competition between *F. hepatica* and *C. daubneyi* in farmed ruminants in Ireland*,* which may influence the epidemiology of infection.

## Results

### Seasonality

**Fig. 1:**
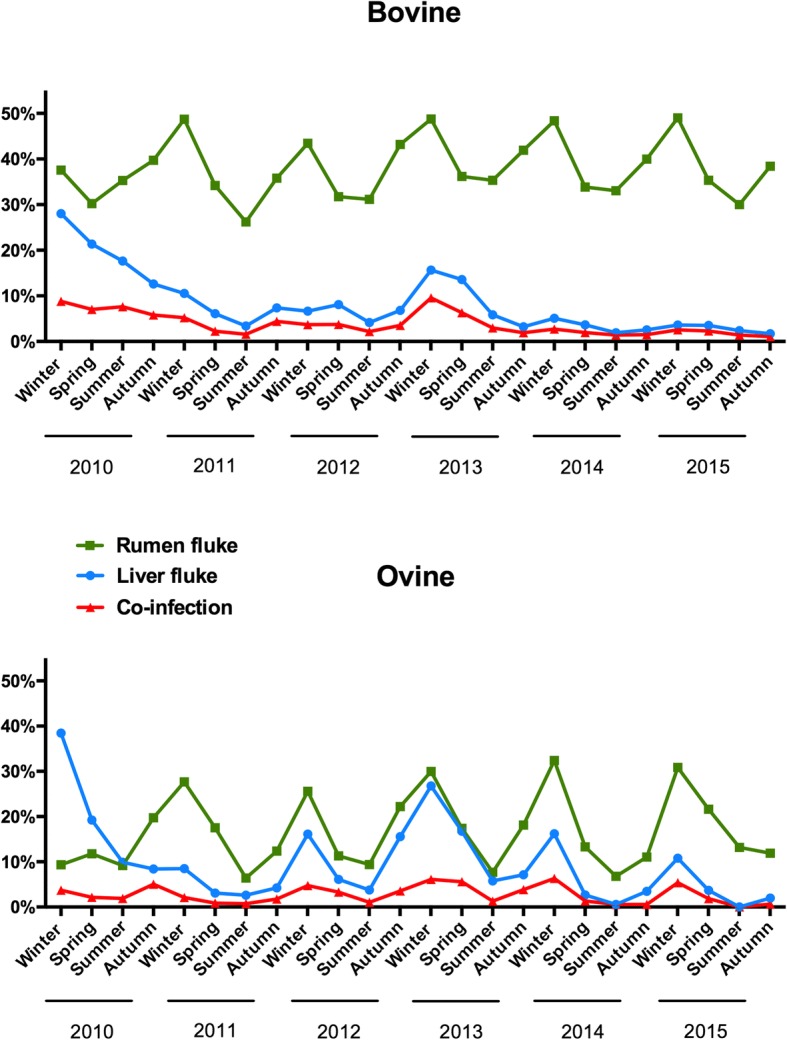
Seasonal distribution of the % positive bovine and ovine faecal submissions to the regional veterinary laboratories from 2010 to 2015. Co-infected submissions are also included in the percentages of each single parasite

Figure 1 shows the seasonality of liver fluke, rumen fluke and concurrent infections in samples from cattle and sheep to RVLs between 2010 and 2015. At any given time of year, rumen fluke infection was more common in cattle than in sheep and generally more frequent than liver fluke infection. In both host species, rumen fluke infection rates peaked during the winter season. In sheep, liver fluke infections followed the same seasonal pattern. In contrast there was no discernible seasonality to patent liver fluke infections in cattle. With respect to bovine cases, winter 2013 had the highest percent positivity for rumen fluke positive submissions (48.8%), while the summer season of 2011 had the lowest (26.2%). In sheep, winter 2014 showed the highest percentage of rumen fluke positive submissions (32.4%), and summer 2011 the lowest (6.4%). During the winter of 2012/2013, a slightly higher percentage of liver fluke infection in both cattle and sheep (15.6% and 26.8%, respectively) was recorded. Overall, the frequency of co-infection with both parasites was similar in cattle and sheep (≤ 10%), and the seasonal pattern resembled that of the liver fluke in each host species.

### Association between *F. hepatica* and *C. daubneyi*

Chi-square test of independence was used to study the relationship between parasite infections in the two host species. The results indicated that if infection with one parasite was considered as a risk factor, there was a positive association between both infections and odds of infection with the other worm. The strength of this association increased from 2010 to 2015: OR from 1.123 in 2010 (*χ*^2^ = 5.697, *df* = 1, *P* = 0.017) to 2.967 in 2015 (*χ*^2^ = 38.395, *df* = 1, *P* < 0.0001) (Table 1). Similar results were obtained when each species was analysed separately (data not shown). In addition, the odds for being positive for rumen fluke infection were shown to be 3 times higher in cattle than sheep (*χ*^2^ = 1027.721, *df* = 1, *P* < 0.0001, OR = 3.134, 95% CI: 2.913–3.371), while the odds for sheep to be positive for liver fluke infection were slightly higher than those for cattle (*χ*^2^ = 29.888, *df* = 1, *P* < 0.0001, OR = 1.288, 95% CI: 1.176–1.410)].

**Table 1:** Association between the two parasites from cattle and sheep submissions

Year	*χ* ^2^	OR	95% CI
2010	5.697*	1.123	1.021–1.235
2011	67.591***	1.931	1.647–2.265
2012	41.394***	1.572	1.368–1.805
2013	41.119***	1.645	1.411–1.917
2014	20.478***	2.159	1.535–3.035
2015	38.395***	2.967	2.071–4.250

*P < 0.05, **P < 0.01, ***P < 0.001

*Abbreviation*: *CI* confidence interval

Chi-square analysis and odds ratio (OR) refer to individual years

### Spatial analysis

**Fig. 2:**
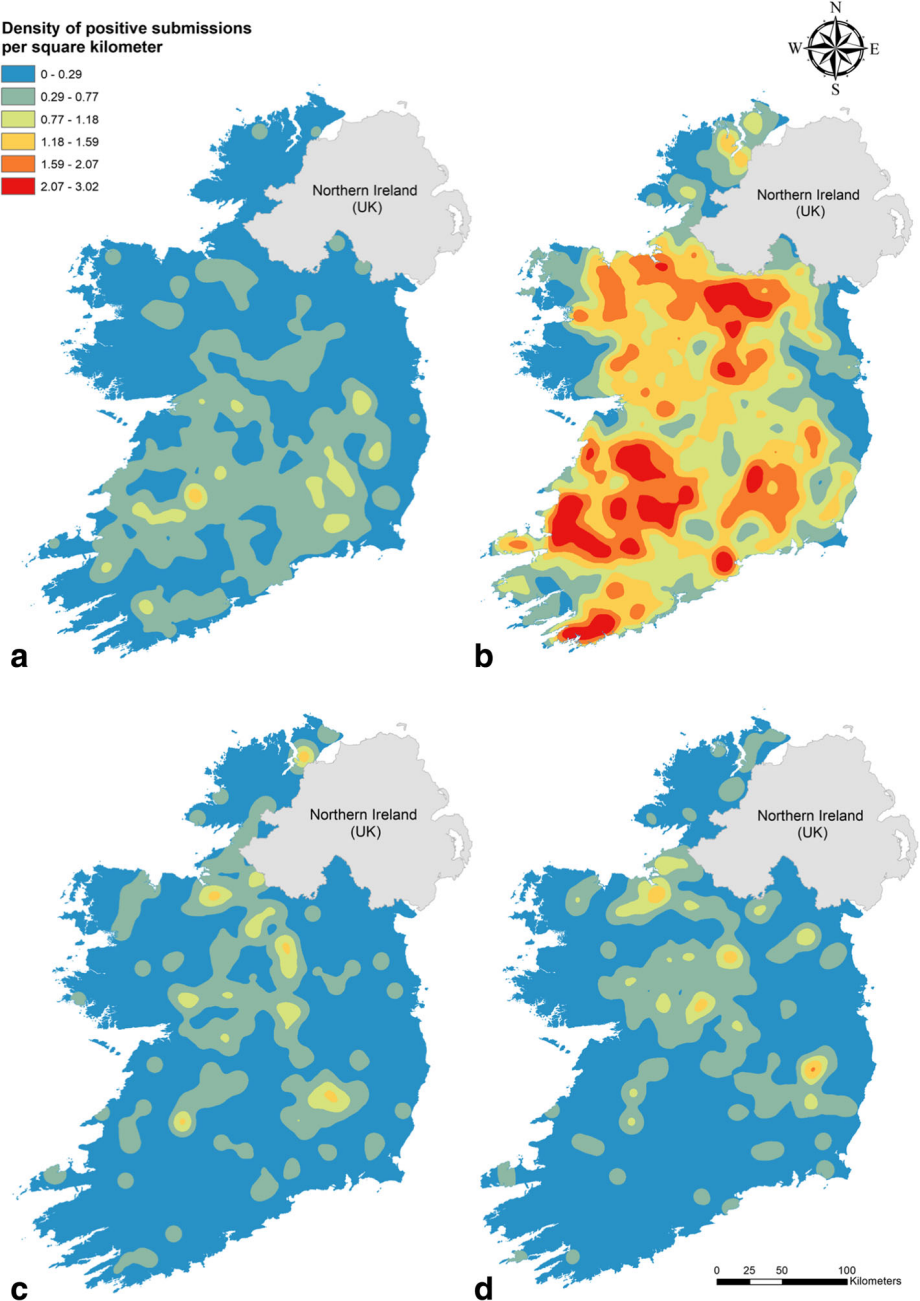
Kernel density analysis of the diagnosed bovine and ovine faecal submissions to the regional veterinary Llboratories from 2010 to 2015. **a** Bovine liver fluke. **b** Bovine rumen fluke. **c** Ovine liver fluke. **d** Ovine rumen fluke

**Fig. 3:**
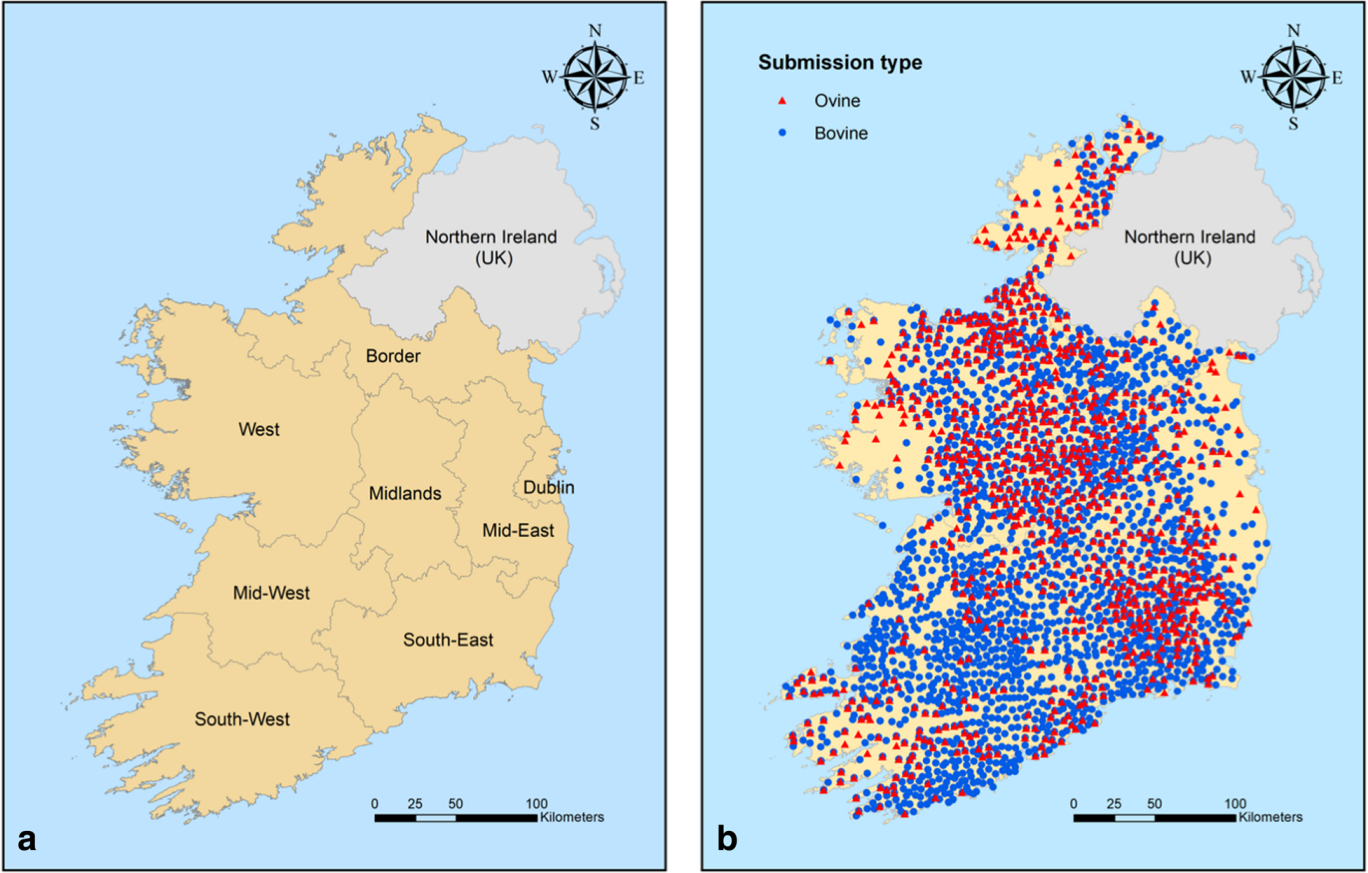
Spatial features of study area and data. **a** Irish regions. **b** DED location of bovine and ovine submissions to the regional veterinary laboratories between 2010 and 2015

**Fig. 4:**
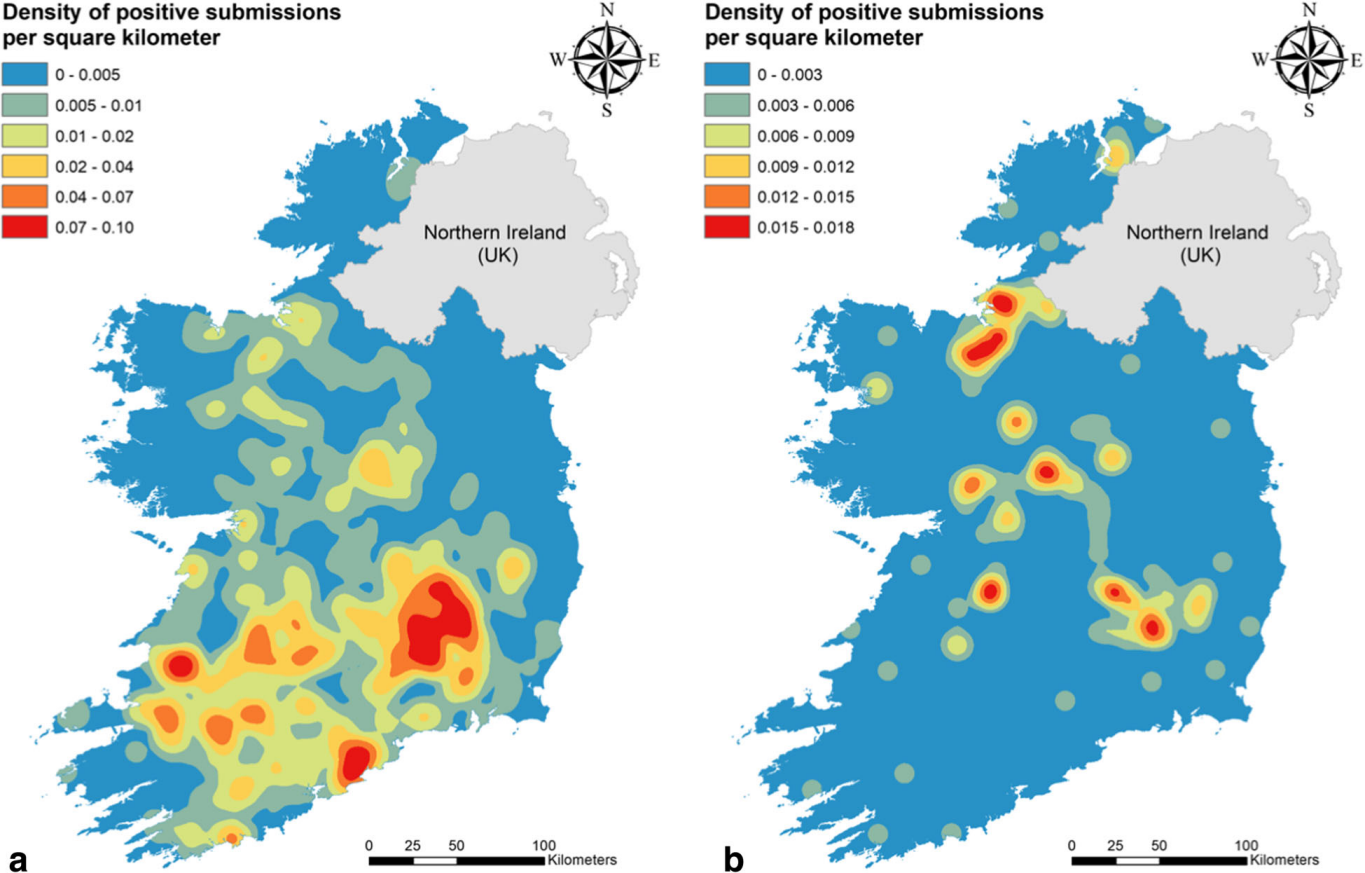
Kernel density analysis of co-infection with liver fluke and rumen fluke in submissions from Irish cattle (**a**) and sheep (**b**)

Figure 2 shows the density distribution of rumen fluke and liver fluke infections in cattle and sheep. Reflecting the characteristic infection rates described above, rumen fluke infections in cattle had the highest spatial density with three main clusters in the Border-Midlands-West, Mid and South-West and South-East (see Fig. 3a for Irish regions). Liver fluke infections were less frequent in the Border-Midlands-West than in the southern regions. In contrast, density maps of either parasite in sheep did not show such a pronounced geographical pattern. Density levels and distribution were similar between both ovine parasitic infections, showing moderate hot spots mainly in the Border-Midlands-West and South-East. The density maps of co-infection mirrored those of the distribution of liver fluke in both cattle and sheep (Fig. 4). Overall, the co-infection map in cattle showed a higher density than in sheep.

### Predicted distribution and risk factors

**Fig. 5:**
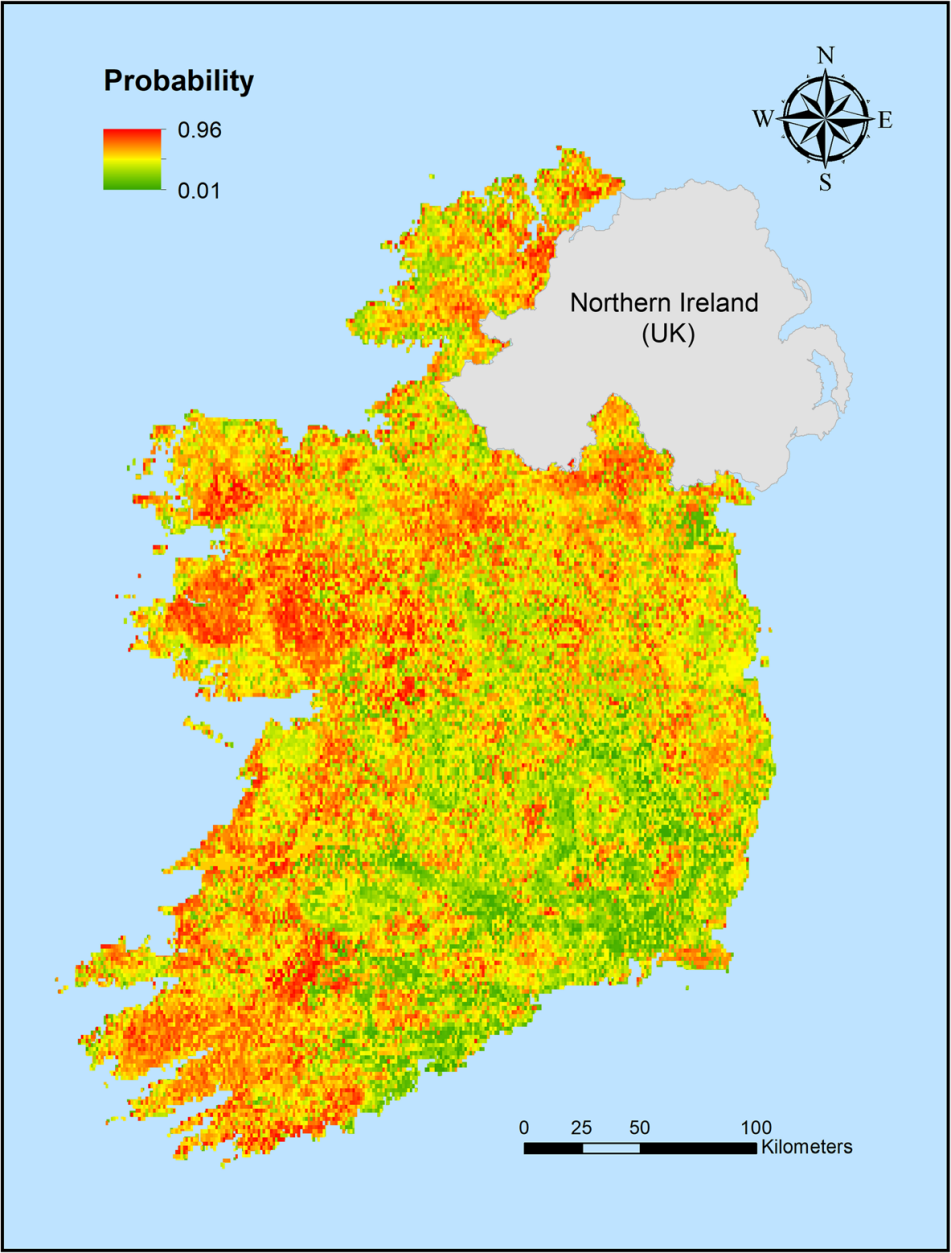
Risk map of rumen fluke infection in Ireland

Figure 5 shows the predicted spatial distribution of rumen fluke infection in Ireland. While probability of infection is highest in the western part of the country and the border with Northern Ireland, the model predicts that infection is generally widespread, also affecting some areas in the east. The only areas with low predicted risk are situated in the south-eastern part of the island. The model performance was assessed by Cohen’s Kappa (0.45) (values of > 0.4 regarded as acceptable), area under the curve (AUC) (0.62), sensitivity (0.65) and specificity (0.57) [23].

The 10 most important variables (in order of importance), according to the Mean Decrease of Gini Index were: soil drainage, land cover, habitat, rainfall in September (for the period 2010–2015), vegetation in September 2014, rainfall in June 2014, vegetation in October 2014, rainfall in May 2014 and vegetation in April and February 2014.

## Discussion

The recent increase in the prevalence of paramphistome infection throughout Europe, including Ireland and Great Britain, has raised the question of how this may affect the development and distribution of *F. hepatica* infection, with which it shares many biological characteristics. This study aimed to analyse the spatial distribution and possible interaction between infection with the two parasites in cattle and sheep in Ireland, where climatological and environmental conditions are particularly favourable for the development of both flukes and their intermediate hosts. The surveillance dataset used in this study was partly based on diagnostic submissions, and may thus have been biased with regard to liver fluke infection, which represents a weakness of the study. However, we believe the size and spatial distribution (Fig. 3b) of the submission data justify their use. Furthermore, as practitioners rarely specifically request testing for rumen fluke due to the apparently low clinical significance of this parasite, it was felt that including surveillance data in the model provided a greater number of true positive outcomes. Inclusion of both cattle and sheep enterprises in the analysis also helped to broaden the scope of the model.

Our results showed consistent winter peaks in rumen fluke percent positive submissions in both cattle and sheep. Similar seasonal fluctuations have also been reported from elsewhere, although the timing of the peaks shifts somewhat depending on specific climatic conditions and the typical length of grazing season [6, 8, 12, 24]. Hatching of parasite eggs and development of the intermediate host are dependent on adequate moisture and temperatures above 10 °C (ideally between 18–27 °C), which occur in Ireland during late spring and summer [21, 25–27]. It can therefore be assumed that encysted metacercariae are present on pasture from late summer, ready to be ingested by the definitive host. Considering a pre-patent period of 3 to 4 months, infected animals will start shedding eggs by the onset of winter [19, 28]. Interestingly, seasonal submissions positive for rumen fluke in cattle rarely fell below 30%, even during the summer months. This may be due to chronic infections with parasites continuing to shed eggs during spring and summer albeit at lower rates (during the winter cattle are not exposed to contaminated pastures) and/or a reduced exposure to new infections in spring due to overwintered eggs or metacercariae. In sheep, the occurrence of rumen fluke infection was overall lower than in cattle throughout the study, but also showed marked seasonality ranging between 32% in winter to 6% in summer. In sheep, both parasites had a similar seasonal variation. In contrast, there was little seasonal variation in liver fluke egg shedding in cattle (Fig. 1).

There are limited treatment options for rumen fluke infection, as there are no commercial preparations with label claims for *C. daubneyi*. The only drug that has consistently been reported to have activity against immature and adult worms is oxyclozanide [29, 30] while there is a single report of closantel being effective after oral administration [30]. Current recommendations in Ireland are that specific treatment for *C. daubneyi* is only warranted if clinical signs are apparent. In contrast, most farming operations treat routinely against liver fluke once or twice a year using a range of products such as nitroxynil, triclabendazole, albendazole, oxyclozanide, clorsulon, closantel or rafoxanide [31]. Dairy cattle generally receive a single treatment during the dry period, at the beginning of housing [32], while beef cattle may receive several treatments. Sheep are much more likely than cattle to suffer acute losses due to the migratory behaviour of immature flukes [33], and also tend to be treated more frequently. However, they generally remain on pasture (potentially exposed to re-infection) until the start of the lambing season (January-February). Consequently, it is possible that seasonality is only observed in ovine liver fluke infection because these hosts spend longer periods on pasture. In addition, resistance to triclabendazole which, due to its efficacy against immature worms has usually been the drug of choice in sheep, has been reported in Ireland [34, 35].

We found that infection rates for rumen fluke were significantly and consistently higher in cattle than in sheep (Fig. 1), with the odds of being positive for rumen fluke eggs about 3 times higher in bovine than in ovine submissions. A significantly higher prevalence of *C. daubneyi* infection in cattle as compared to sheep has also been reported from veterinary surveillance datasets, diagnostic datasets and abattoir surveys in Ireland [36], the Netherlands [37], Italy [38] and the UK [39] suggesting that either cattle are more susceptible to infection, or have a greater level of exposure. An argument that supports the susceptibility hypothesis is that during experimental infections with *Calicophoron microbothrium*, it was shown that, in cattle, the worms had a shorter prepatent period, survived migration better, reached larger sizes, and produced eggs for a longer period when compared to C. *microbothrium* infections in either sheep or goats [40]. Whether a similar situation occurs with regard to *C. daubneyi* is yet to be determined. One argument against the greater exposure hypothesis is the longer winter housing period typical in cattle farming.

As in previous studies [12, 20, 37] we found a strong association between infection with the two parasite species in both sheep and cattle. Considering the shared intermediate host this is hardly surprising. What was not expected was the gradual increase in the likelihood of co-infection from odds ratios of about 1.1 to 3.0 over the course of the 6-year study period. Therefore, as far as the vertebrate host is concerned there seems to be no competitive interaction between the two parasite species. In fact, considering their respective migratory routes and infection sites this would seem highly unlikely in any event. In contrast, co-infected snails are relatively rare probably due to a mutual antagonism/competition between the fluke species and/or reduced survival of co-infected snails [41–43]. For example a recent epidemiological investigation of a severe rumen fluke outbreak in Ireland found only 1 out of 70 snails collected from pasture infected with *F. hepatica*, 39 with *C. daubneyi* while none were co-infected [11].

The spatial analysis of our data revealed three clusters of rumen fluke infection in cattle which coincide with the areas in Ireland where most cattle herds, both dairy and beef, are located (South and Mid-West, South-East, and the border with Northern Ireland) [44] (Fig. 2b), indicating that both beef and dairy production systems favour infection with paramphistomes. In contrast, density values (positive herds per square km^2^) for liver fluke infection in cattle are much lower (average values of 1.13 for rumen fluke infection and 0.3 for liver fluke infection), with foci concentrated chiefly in the southern part of the country, where most dairy enterprises are located (Fig. 2a). There are a number of potential factors which may explain this finding including a longer grazing period and higher herbage intake in the diet, reduced anthelmintic product range, and reduced frequency of anthelmintic treatment in dairy compared to beef cattle [45–47] arising from restrictions on use during lactation. It is therefore possible that control measures against liver fluke in beef herds are not only more effective, but also that these herds may have a lower exposure to contaminated pastures. In sheep flocks, liver fluke and rumen fluke share similar density levels (average of 0.2 for both infections), and hot spots ranging from the north-west, through the midlands to the south-east. Kernel density analysis of co-infection in both host species (Fig. 4) showed similar patterns to those of liver fluke infections (Fig. 2a, c) probably because the presence of the liver fluke was the limiting factor. However, density profiles in cattle were more pronounced. The reason for this may be that as the number of cattle herds in Ireland is much greater than that of sheep flocks (110,995 and 32,111, respectively, in 2010) [44], positive DED were situated closer together, resulting in higher density calculations. Given that Kernel density magnitude calculations depend on both the value of percent positive submissions at each DED, and its distance to the surrounding positive neighbours, higher values will be obtained if more positive neighbours are present.

We show here the first spatial model of rumen fluke infection in Ireland. According to this model some potential risk factors for rumen fluke infection were similar to those identified in previous spatial models of *F. hepatica* infection in Ireland [48, 49] and included vegetation and rainfall variables. This reflects the reliance of both parasites on the same intermediate snail hosts as well as similar environmental requirements of the free-living life-cycle stages. On the other hand, there were some factors, such as soil drainage, land cover and habitat, that were not among the potential risk factors for *F. hepatica* but were of major importance for *C. daubneyi* infection. These environmental factors were also found to be of primary importance in previous rumen fluke models from Italy, Spain and Wales [39, 50, 51]. The relevance of these variables is not surprising as soil drainage will affect the moisture level and consequently the development of the snail intermediate host. Moreover the presence of the definitive and intermediate hosts are both directly linked to land use and habitat. In addition, these last two variables may be of relevance for the distribution of wild ruminants. However, while both *F. hepatica* and *C. daubneyi* occur in wild ruminants in Ireland, they are not thought to represent a significant source of infection [52]. In contrast, temperature, which was the most important variable in a liver fluke model based on the same study period [49], was not among the main 10 risk factors in the rumen fluke model. This may be due to the fact that the shedding pattern of *F. hepatica* cercariae from snails is more sensitive to temperature than *C. daubneyi*, which is better adapted to daily temperature changes [53]. ‘Slope’ which was also listed as a major factor in some previous studies [50, 51, 54] (i.e. the probability of infection increased with increasing slope) could not be included in our study because the data layer was not available. The closest proxy, elevation, though not among the main risk factors, was associated with a predicted (though not elevated) risk of rumen fluke infection (Fig. 5). Again this was not the case in the predicted distribution of *F. hepatica* [49] which may also be a reflection of subtly different temperature requirements of the larval stages of the two parasites.

Several management factors such as production system, breed, animal density and age group have been suggested to affect the prevalence and fluke burden of *C. daubneyi* in cattle although frequently with inconsistent results [14, 36, 51]. Unfortunately management data were not available for most of the cattle farms included in this study and were therefore not analysed. The sheep survey dataset we employed in our study was provided by Martinez-Ibeas et al. [55], who identified lowland pastures, mixed grazing and the Suffolk breed as risk factors for *C. daubneyi*.

It is interesting to note that when both cattle and sheep farms are included in a study, the relevance of cattle in the epidemiology of *C. daubneyi* is evident. Jones et al. [42] found that the total number of cattle or number of heifers or steers present on a farm were positive predictors in models independently developed for cattle, sheep or all farms together. Rumen fluke models showed that average number and timing of treatments against *F. hepatica* are positive predictors for *C. daubneyi* infection [39]. As the authors suggest, this finding may be explained by the removal of competition with *F. hepatica* as a result of selective control measures, allowing *C. daubneyi* to spread more freely. Other management factors such as herd size or length of grazing season that have been shown to be of importance in the epidemiology of *F. hepatica* [56–60] may also influence *C. daubneyi*.

## Conclusions

In conclusion, there are many biological commonalities between liver and rumen fluke life-cycles and environmental preferences, but also some subtle yet important differences which affect their occurrence, seasonality and risk factors for infection. Two of the main factors that determine the epidemiology of infection with both parasites are themselves undergoing change. The first of these is the declining efficacy of *F. hepatica* control measures due to increasing resistance to triclabendazole; the second is global climate change, which is predicted to extend the season of *F. hepatica* in northern Europe [61] and is likely to have a similar effect on *C. daubneyi*. Against this shifting background, it remains to be seen whether the dynamics of these two trematodes will reach an equilibrium, and if the clinical consequences in livestock will change. To further investigate the interactions between both parasites, (co-)infection trials in both the intermediate and definitive hosts are required. In addition, further work is needed to elucidate the effects of certain management factors such as treatment schedules and specificity. Finally extending the rumen fluke predictive model to European level will help to determine environmental and climatological factors more broadly by involving a greater variety and range of conditions. This will also allow for a more comprehensive comparison with predictive models for liver fluke which have already been developed.

## Methods

### Infection status data

#### Veterinary surveillance data

Bovine and ovine liver fluke and rumen fluke data, based on faecal examination of diagnostic samples submitted between 2010 and 2015, were provided by the Department of Agriculture Food and the Marine (DAFM) through its Regional Veterinary Laboratories (RVLs). All samples were analysed by faecal sedimentation [62]. A total of 48,886 submissions were included for analysis. Although the precise reason for submission was not recorded in all cases, most were made on clinical grounds. Approximately 18% of submissions explicitly stated a suspected aetiology of fluke infection while in the remainder veterinarians had requested a general parasitological faecal exam in addition to other microbiological tests. Furthermore, a small proportion (*c.*5%) of submissions was made for herd health screening purposes (submissions in which 9 or more faecal samples were supplied). Finally, about 10% of submissions resulted from animals sent to the RVLs for *post-mortem* examination.

#### Sheep flock survey data

In addition to the passive surveillance records, data collected during a stratified nationwide survey of 290 sheep flocks between October 2014 and February 2015 were also included in the rumen fluke model [55] (Flukeless research project, Research Stimulus Fund, Project no. 13/S/405). During the survey a standardised sampling kit was posted to participating farmers with a request for fresh faecal catch samples from 20 mature ewes. The group of participating farmers represented the national geographical spread of sheep flocks in Ireland according to the Irish Census of Agriculture (2010). Presence and absence of *F. hepatica* or paramphistome eggs was determined by the sedimentation technique [62]. Flocks where at least one rumen fluke egg was observed were considered positive.

### Seasonality calculations

Submissions to the RVLs were used for this analysis. Percent positive submissions for liver fluke, rumen fluke and co-infection were calculated separately for submissions in Spring (March, April, May), Summer (June, July, August), Autumn (September, October, November) and Winter (December, January, February) for ovine and bovine submissions between 2010 and 2015. Submissions positive for both, *F. hepatica* and *C. daubneyi* (or *Paramphistomum leydeni*), were also included in the single-infection analyses for each of the parasite species. Submissions for each season were extracted from the dataset as described below.

### Associations and risk estimates

Ovine and bovine submissions to the RVLs were analysed together as well as separated by species, to examine a possible association between the two parasites. In addition, the relationship between host species and parasite infection was explored by Chi-square test of independence and odds ratio (IBM SPSS Statistics software version 20).

### Spatial analysis

The RVLs dataset was employed in this section. To ensure precise GPS coordinate mapping, only data with accurate herd numbers were included in the spatial analysis reducing the number of analysed submissions to 28,303. Spatial analyses were based on percent positive submissions to each District Electoral Divisions (DED). The centroids of DED locations of all submissions are shown in Fig. 3b. The data were collapsed by DED using IBM SPSS version 24, the table exported to MS Excel (MS Office version 16.8) and percent positive submissions for liver fluke, rumen fluke and co-infection in each DED were calculated.

Kernel density analysis was applied to create ‘heat maps’ of each parasitic infection in both cattle and sheep using the ‘Kernel density’ tool in ArcMap 10.4 (ArcGIS © ESRI Redlands, CA). This tool calculates the density of features in a neighbourhood around those features. The function is based on the quartic kernel function described by Silverman [63]. Briefly, the density at each output raster cell was calculated by adding the values of all kernel surfaces where they overlaid the raster cell centre. The magnitude at each DED centroid was distributed throughout 15 square km (average DED area), and a density value was calculated for each cell using the planar method. A mask of the Irish map was used for visualization purposes. In order to facilitate better comparison of the density distribution of the two parasites in cattle and sheep, the same scale and legend were used on all maps. Maps for bovine and ovine co-infection densities were also generated.

### Modelling

In order to comply with data protection requirements, modelling was performed in The Centre for Veterinary Epidemiology and Risk Analysis (CVERA) at UCD.

#### Response data

The spatial predictive model of rumen fluke infection was based on both surveillance data and ovine survey data, both collected between October 2014 and February 2015. Farm GPS locations of submitted samples to the RVL were extracted and the ovine survey data (which already included location information) were merged. Overall, presence or absence of infection from 477 herds and 351 flocks were included in the model. All cattle herds plus 61 sheep flocks belonged to the RVLs dataset, while the remaining 290 sheep flocks’ data derived from the cross-sectional sheep survey. Since veterinary advice in Ireland is not to treat routinely for rumen fluke infections, we hypothesised that faecal examination data gave a truthful representation of infection. While the sensitivity of the sedimentation technique for the detection of rumen fluke infection is unknown, it is assumed to be similar if not higher (due to the lack of routine treatment and therefore higher numbers of eggs per gram expected, as well as the fact that eggs are not secreted in the gall-bladder) to that for fasciolosis, which produces eggs of similar size and shape. The sensitivity for detecting *F. hepatica* infection using the sedimentation method can range 43 to 64% in cattle depending on the amount of faeces employed, but is thought to be higher in sheep due to lower variability in egg shedding and smaller volume of faeces produced. Specificity of the sedimentation method is generally estimated to exceed 95% [64, 65].

#### Predictor variables

Variables used to develop the model and their sources are listed in Table 2. Rainfall and temperature datasets comprised data collected in 25 meteorological stations, and interpolated to cover the whole area of the country. Vegetation variables (NDVI and EVI) are numerical indices based on wavelengths reflected by the vegetation, obtained by remote sensing (RS) or satellite imaging, used to compute values that quantify plant biomass and/or vigour. Habitat, land cover and soil-related variables are categorical variables.

**Table 2:** Data-layers included in the geographical information system (GIS) for modelling

Variable	Description	Source and resolution
Period 2010–2015 climatic variables	Averages of annual, seasonal and monthly mean temperatures (°C), total rainfall (mm), and annual total number of rain-days (daily rainfall ≥ 0.2 mm)	Met Éireann (1 × 1 km), interpolated values
Year 2014 climatic variables	Averages of annual, seasonal and monthly mean temperatures (°C), total rainfall (mm), and annual total number of rain-days (daily rainfall ≥ 0.2 mm)	Met Éireann (1 × 1 km) Interpolated values
Soils, subsoils and soils drainage	National Soils Database	EPA (scale 1:250,000)
Habitat	National Habitat Indicator Map	Teagasc (25 × 25 m)
Land cover	2012 CORINE land cover dataset	EPA (25 ha minimum mapping unit)
Elevation	National Elevation Map	UCD Maps and GIS library. Processed by CVERA (25 × 25 m)
Vegetation	Monthly normalized difference vegetation index (NDVI) and enhanced vegetation index (EVI) from 2014	Avia-GIS (250 × 250 m)

*Abbreviations*: *EPA* Environmental Protection Agency; *UCD* University College Dublin; *CVERA* Centre for Veterinary Epidemiology and Risk Analysis

All data files were projected to the WGS1984 geographical coordinate system and converted to raster file. All final rasters had the same cell size.

#### Random Forest methodology

First, the spatial features of sheep flocks were investigated to establish the best method to extract predictor variables. This was done because in Ireland some sheep flocks use shared grazing areas called commonages. These are pieces of land, often in rugged terrain, where farmers have traditional ‘grazing rights’. In many cases, commonages can be located several kilometres away from the farm. To establish the situation of the flocks included in our dataset, commonage parcels available for grazing by each flock were investigated by matching herd numbers with the DAFM’s Land Parcel Identification System (LPIS) database. The mean area of the farms was 0.52 km^2^ (standard deviation, SD: 0.43), and mean area of commonage parcels was 3.67 km^2^ (SD: 7.7). Distance between the farms and commonages was calculated by using the geoprocessing tool ‘Near by group’ in ArcMap. Eighty flocks out of 351 had common grazing rights. Of those, only 9 were more than 1 km distant from the commonage parcel. The mean distance from each farm to their assigned commonage was 0.75 km (SD: 2.9) (range: 0 to 19.47 km). It should also be pointed out that most Irish farms do not exceed 1 km^2^ of agricultural area utilised (AAU) [44].

The shapefile containing geographical coordinates and presence/absence information (1/0) was projected to the WGS1984 geographical coordinate system using ArcMap. The response data was first ‘balanced’ by creating an even number of presence and absence points, to avoid a biased prediction. Then, predictor values at each farm location were extracted using a buffer of 1 km^2^ around each farm to allow for the distance of some commonages (see above) and mean farms areas. Using the ‘Zonal Statistics as Table’ tool in ArcMap, average values were calculated for numerical variables, while majority values were used in the case of categorical values.

The predictive model was developed using VECMAP version 2.2.17186.1461 (Avia-GIS) following the same approach as described by Lucena et al. [49]. Briefly, Random Forest (RF), a machine learning algorithm that uses classification and regression trees (CART), was used to model rumen fluke infections in cattle and sheep [66, 67]. The trees are constructed using a random subset of both the data points of presence/absence of infection and the explanatory variables recorded for each data point. The RF model has the advantage that it reduces bias in the training set while providing an indication of the importance of each variable. For each tree, a subset of the data which is excluded from building the model (‘out-of-the-bag’, OOB), is used for validation purposes. Initially, a model was developed to select significant variables to cluster the data. Then, to improve model performance, a variable reduction forest was performed. The final RF was created by including the 50% subset of the most important variables.
